# Exploring the Associations between Early Childhood Development Outcomes and Ecological Country-Level Factors across Low- and Middle-Income Countries

**DOI:** 10.3390/ijerph18073340

**Published:** 2021-03-24

**Authors:** Kasim Allel, Gerard Abou Jaoude, Stavros Poupakis, Neha Batura, Jolene Skordis, Hassan Haghparast-Bidgoli

**Affiliations:** Institute for Global Health, University College London, London WC1E 6BT, UK; gerard.jaoude.15@ucl.ac.uk (G.A.J.); s.poupakis@ucl.ac.uk (S.P.); n.batura@ucl.ac.uk (N.B.); j.skordis@ucl.ac.uk (J.S.); h.haghparast-bidgoli@ucl.ac.uk (H.H.-B.)

**Keywords:** early childhood development, low- and middle-income countries, inequalities, Sustainable Development Goals, child health

## Abstract

A poor start in life shapes children’s development over the life-course. Children from low- and middle-income countries (LMICs) are exposed to low levels of early stimulation, greater socioeconomic deprivation and persistent environmental and health challenges. Nevertheless, little is known about country-specific factors affecting early childhood development (ECD) in LMICs. Using data from 68 LMICs collected as part of the Multiple Indicator Cluster Surveys between 2010 and 2018, along with other publicly available data sources, we employed a multivariate linear regression analysis at a national level to assess the association between the average Early Childhood Development Index (ECDI) in children aged 3–5 and country-level ecological characteristics: early learning and nurturing care and socioeconomic and health indicators. Our results show that upper-middle-income country status, attendance at early childhood education (ECE) programs and the availability of books at home are positively associated with a higher ECDI. Conversely, the prevalence of low birthweight and high under-5 and maternal mortality are negatively associated with ECDI nationally. On average, LMICs with inadequate stimulation at home, higher mortality rates and without mandatory ECE programs are at greater risks of poorer ECDI. Investment in early-year interventions to improve nurturing care and ECD outcomes is essential for achieving Sustainable Development Goals.

## 1. Introduction

Children’s well-being and development are central themes of the United Nations Sustainable Development Goals (SDGs) which focus on the achievement of a better and more sustainable future for all. Children’s well-being and development are explicitly targeted by Goal 4 (quality education), which aims to ensure that all children have access to quality early childhood development, care and pre-primary education. Likewise, these themes are also targeted by several other goals, such as poverty reduction, health and nutrition, gender equality and ending violence [1,2]. Every child should survive, thrive and develop to his or her full potential, particularly during the first five years of life, which is essential for children’s growth and lifelong health and well-being. Effective early childhood development (ECD) programmes have been proven to improve nutrition, health status, cognition, socioemotional status and language proficiency [3]. Collectively, these gains can reduce disparities throughout the life-course [3,4,5,6,7]. Thus, the early years shape individuals’ developmental trajectory and interventions in this life stage generally comprise cost-effective strategies to promote human development while decreasing criminality and rates of behaviour disorders [8,9,10].

Even though poor childhood development, including developmental delay, is of global concern, low- and middle-income countries (LMICs) are more likely to have poorer ECD outcomes on average [11]. Children from poorer families are less likely to receive adequate stimulation. They are exposed to constant environmental and economic challenges, such as poor sanitation and hygiene, food insecurity, deficient living conditions, poor health and social deprivation [12]. In the long term, these socioeconomic differences negatively impact adult life [8,10,13]. There is a need for low-cost, effective and scalable interventions in children’s early years in LMICs. However, such interventions cannot be effectively designed and implemented without understanding how children’s environment interplays with ECD.

Prior research has posited the existence of three environments where cultural, biological and social determinants interact to shape children’s development and health outcomes [14,15,16,17,18]. First, the household and family represent the child’s microsystem where parental background, material deprivation and living conditions play a vital role in developing the child. Second, the community and neighbourhood represent the mesosystem where exposure to nature and contamination, safety and accessibility to social services may impact development. Third, the macrosystem involving the sociopolitical environment, including broader cultural and regulatory aspects, affect ECD. Some studies have separately investigated the effect of the micro- and meso-level factors on ECD [3,14,16,19]. They have found that nurturing care and protection acquired from parents, family and society vastly improve ECD at either the household or community levels. However, these findings are related mainly to high-income countries and do not incorporate further analyses using national-level data. Few studies have described ECD using national-level estimates, including some that use the World Health Organization (WHO) Nurturing Care Framework [20,21,22], and only one of them employed a consistent and wide sample of LMICs to describe how the average level of inequalities is linked to ECD across countries [20]. That study did not account for exogenous variables, and only analysed four potential ECD risk factors nationally: (1) stunting, (2) poverty, (3) home stimulation and (4) early childhood education (ECE) attendance. To address this evidence gap, the analyses presented here explore the association between ecological country-specific factors and ECD in 68 LMICs, taking a broader view of possible risk factors compared with previous literature and combining publicly available data from different sources to facilitate the inclusion of a greater number of countries.

## 2. Materials and Methods

### 2.1. Data Sources and Sampled Countries

The study uses cross-sectional data primarily from the Multiple Indicator Cluster Survey (MICS). Our analysis draws on a sample of 68 LMICs with available data on the Early Childhood Development Index (ECDI) at the aggregate level between 2010 and 2018 (Supplementary Materials, Tables S1 and S2) [23]. Values from the most recent year available were used. Additional country-level data used as covariates (Table 1) were extracted from publicly available sources such as the World Bank (WB) databank, the United Nations (UN) human development report and world population prospects, the Demographic and Health Survey (DHS), the World Health Organization (WHO) low birthweight estimates the Global Acquired Immune Deficiency Syndrome (AIDS) Monitoring, and the Joint United Nations programme on AIDS (UNAIDS) 2019 estimates. Country data, including data on nurturing care and early learning, and socioeconomic and health statuses, were obtained for the range of years between 2014 and 2019 depending on availability.

### 2.2. Dependent and Independent Variables

Table 1 displays the description and rationale for each variable used in the analysis and their corresponding sources. The ECDI is a caregiver-reported index that encompasses the development status of children age 36–58 months within four domains: literacy, physical, numeracy and social–emotional development. Being on track refers to achieving between 11 and 15 milestones in each ECDI domain depending on age [25]. The ECDI and the percentage of children developmentally on track were used as dependent variables in separate analyses. All the dependent and independent variables are aggregated at the national level.

Early learning and nurturing care (attendance at ECE, early stimulation at home, children’s books at home and children playing games at home), socioeconomic variables (gender inequality, income group, political stability, net migration rate) and health data (under-5 stunting, low birth weight, pregnant women receiving Human Immunodeficiency Virus (HIV) treatment, maternal mortality, under-5 mortality) were included as independent variables. Full details on the data sources, description of variables and justification for inclusion in the analysis are found in Table 1.

The percentage of missing data for our selected independent variables was around 15%. We imputed missing observations using a multivariate normal regression (MVN) with 50 repetitions. No differences were observed in the average values between the original and imputed variables using corresponding *t*-tests (Table S3). We used variables with full information to impute missing data. These variables included the prevalence of stunted children under the age of 5, under-5 mortality, children’s attendance at early childhood education (ECE) programs, political stability, population size, Human Development Index (HDI) and gross domestic product (GDP) per capita in 2018.

### 2.3. Statistical Analysis

We employed two independent multivariate linear regression analyses to test separately whether country-level factors were associated with two variables: (i) ECDI and (ii) the percentage of children developmentally on track. Both dependent variables were normally distributed (Supplementary Materials, Figure S1).

We based our independent variable selection on the main determinants of ECD reported in the literature, with particular attention paid to the “Country Profiles for Early Childhood Development” report by the United Nations International Children’s Emergency Fund (UNICEF) [25]. These ECD determinants include country-level data on the facilitating environment (e.g., international conventions, policies to ensure social protection and development), threats to ECD (e.g., maternal mortality, poverty level, young motherhood, low birth weight, stunting, inadequate supervision) and the five components of the Nurturing Care Framework: health, nutrition, early learning, responsive caregiving and security and safety.

The final variables used as covariates in the regression analyses were chosen from the previously mentioned ECD determinants following a three-step procedure. First, based on the calculated bivariate correlation between the ECDI and ECD determinants using the Pearson’s coefficient (Supplementary Materials, Figure S2) and joint significance tests, we decided to keep the variables with a moderate or strong correlation (coefficient greater than 0.5) [26]. However, we did not drop under-5 stunting prevalence even though it did not reach the threshold defined above. Stunting is considered as one of the main determinants of ECD, particularly in highly unequal countries and LMICs [17,27]. Third, we combined the chosen variables in a multiple regression analysis using stepwise techniques (backward elimination) with a significance level below 0.2 to achieve better goodness of fit. We also excluded some variables such as GDP and HDI that presented multicollinearity issues (variance inflation factor (VIF) > 10). Finally, we fit our model to the data by employing linear regression analyses with heteroscedasticity-robust standard errors. We examined our regression results to address the linear regression model’s main assumptions by testing our skewness, kurtosis and heteroscedasticity estimates. The results of these tests suggested that our dependent variables were normally distributed and the linear approach employed was therefore correct (Supplementary Materials, Table S5). Figure S3 presents the normal density plot for the regression analysis residuals (model 1), showing random results.

All statistical analyses were conducted using Stata 15 software, MP version 2017, StataCorp LLC., College Station, TX, USA.

## 3. Results

### 3.1. Descriptive Statistics

Our sampled countries represent every WHO region and WB income group except for high-income countries (Table 2). Countries’ socioeconomic and demographic characteristics vary notably by World Bank region (Supplementary Materials, Table S4). Compared with other regions, countries from Africa and South Asia have lower average Gross National Income (GNI) per capita, education level, life expectancy and access to basic sanitation and drinking water. Table 3 presents descriptive statistics for the variables used in these analyses. ECDI and the percentage of children developmentally on track across the 68 LMICs are 73.48 (SD = 14.8) and 72.58% (SD = 14.5), respectively.

Our sampled countries have a high percentage of children with low birth weight (mean = 11.3%, SD = 4.73) and stunting (mean = 21.46%, SD = 13.2). On average, 35.84% of children aged 3–5 years attended education programs, and 23.49% have more than three books at home. On average, 65.13% of the sampled countries exhibit proper stimulation of children at early ages at home (SD = 22.38). Most countries score negatively on political stability and have high levels of gender inequality.

**Figure 1:**
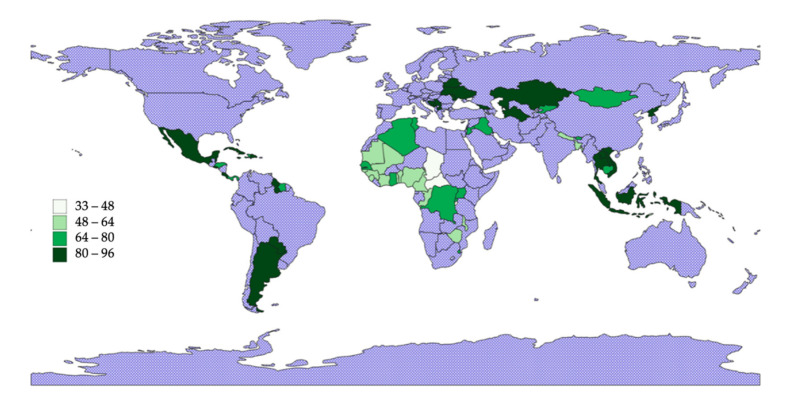
Early Childhood Development Index (ECDI) levels in the sampled countries (*N* = 68 LMICs). Note: Blue area stands for either missing or high-income countries.

**Figure 2:**
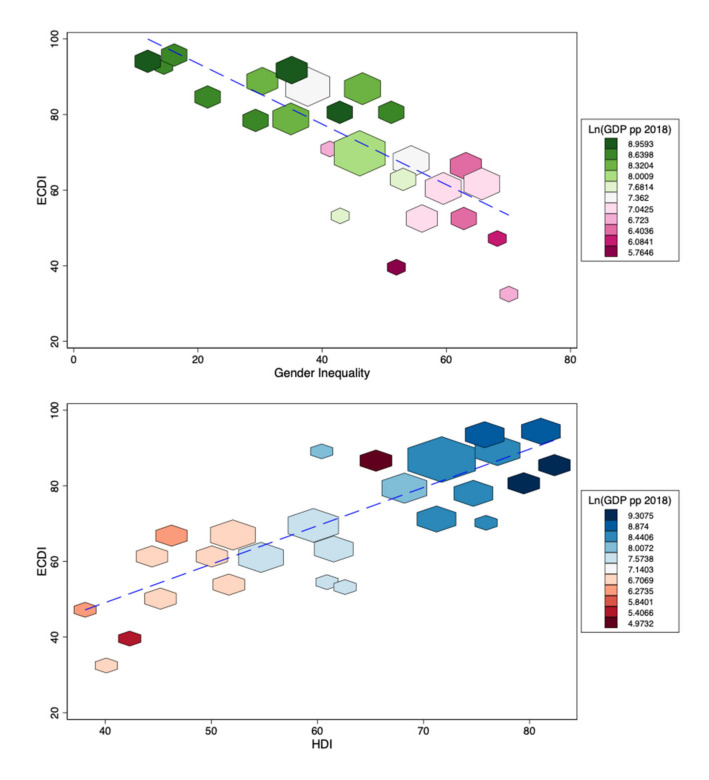
Human Development Index (HDI) and gender inequality rates by ECDI levels (*N* = 68 LMICs)**.** Note: Blue dashed line represents a linear fit between the two variables. Bigger shapes indicate a higher proportion of data points.

**Figure 3:**
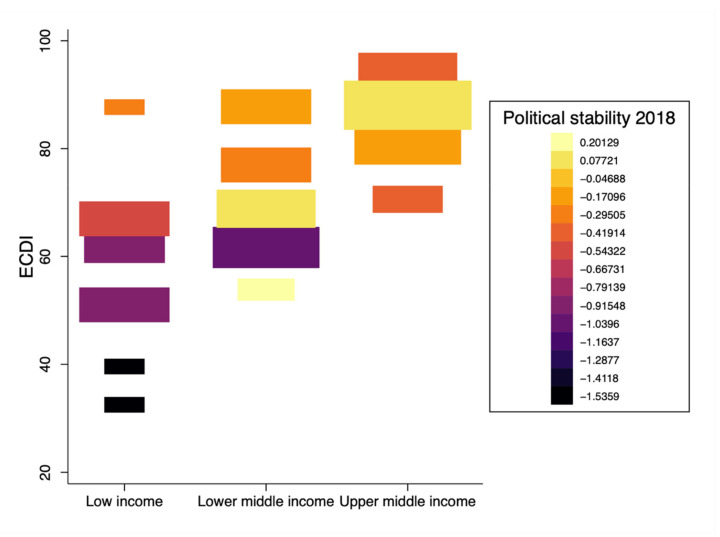
Early Childhood Development Index (ECDI) levels by income category and political stability (*N* = 68 countries). Note: Bigger squares indicate a higher proportion of data points. Y-axis displays country classification by income groups according to the World Bank.

**Figure 4:**
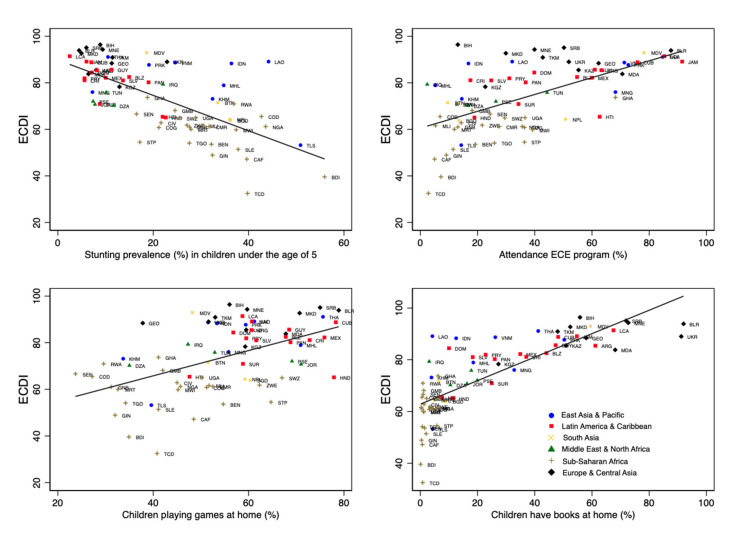
Descriptive results by country (*N* = 68 LMICs). Note: Solid black lines show the linear fit between variables on the y- and x-axes.

There is a considerable variation in ECDI between different regions (Figure 1). Latin American, European and Asian countries have relatively higher ECDI values, with Bosnia and Herzegovina and Serbia benefiting from the highest ECD values in this group. East and Central African countries have the lowest ECDI values, with Burundi and Chad challenged by the lowest ECDI values in this group. Higher gender inequality is observed in poorer countries with lower ECDI values (Figure 2). Similarly, higher ECDI values are observed in wealthier countries with higher HDI values. LMICs with lower political stability have lower ECDI values (Figure 3). Figure 4 shows the relationship between ECDI and four variables: (1) stunting prevalence, (2) attendance at ECE programs, (3) children playing games at home and (4) children having three or more books at home [23]. Higher prevalence of stunting, predominantly in African countries, was negatively associated with ECDI. In contrast, larger numbers of children with three or more books at home and children attending ECE programs were positively associated with greater ECDI. There is a substantial variability across countries in the four variables investigated in Figure 4, but countries in the African region (e.g., Burundi and Chad) had the least favourable values for any variable.

### 3.2. Regression Analysis 

Table 4 displays the linear multivariate analyses to understand how health-related, nurturing care and socioeconomic country-level factors are associated with ECDI (Model 1) and the proportion of children developmentally on track (Model 2).

Model 1 shows that, at a national level, income group, maternal and under-5 mortality rates, percentage of attendance at ECE, percentage of children with books at home, the prevalence of low birth weight and percentage of pregnant women receiving HIV treatment were statistically significant predictors of ECDI. An increase of one unit in countries’ under-5 mortality rate is associated with a decline of 1.42 points in the national ECDI (*p*-value < 0.001). Similarly, maternal mortality had a similar association in terms of the coefficient’s direction but was smaller in size (*p*-value = 0.01). In contrast, upper middle-income countries were associated with an increase in ECDI of about 7.17 points (*p*-value = 0.04), compared with low-income countries. The two aggregated variables of nurturing care and early learning had a positive link with ECDI (*p*-value = 0.01 for ECE program attendance, and *p*-value = 0.04 for children’s books at home). Finally, higher percentages of low birthweight had an inverse association with ECDI across countries (*p*-value = 0.08).

The same independent variables from Model 1 were statistically significant in Model 2, except for the national percentage of children with books at home and low birth weight prevalence. Upper middle-income countries were linked to a greater developmentally on-track score of 8.33 points than low-income countries (*p*-value = 0.02). Countries’ under-5 mortality rate had a stronger linkage with the developmentally on-track score than with ECDI in Model 1 (*p*-value < 0.001). The coefficients of aggregated maternal mortality and attendance at ECE programs were the same as the preceding model. Surprisingly, the higher the percentage of pregnant women receiving HIV treatment nationally, the lower the ECDI and developmentally on-track scores (*p*-value_Model1_ < 0.001, *p*-value_Model2_ < 0.001, respectively).

## 4. Discussion

In this study, we pooled publicly available data from several sources to analyse how different country-level variables are associated with ECD in 68 LMICs. Using linear regression methods, we examined how socioeconomic, nurturing care and early education and health and mortality-related indicators affect ECDI and the prevalence of children developmentally on track, nationally. To our knowledge, this is one of the first studies using aggregated data at a national level.

The figures shows that LMICs in Central and Eastern Africa have the lowest ECDI scores, while European and Central Asian LMICs have the highest. Our analyses show that improved nurturing care and early education (i.e., attending ECE programs and children’s books at home) and country income level are positively associated with ECD, while maternal and under-5 mortality negatively affect ECD.

These findings are consistent with those reported by previous studies [20,22,25,27,28,29,30,31,32,33]. Maternal and under-5 mortality rates are country-level variables with a strong link to ECD and reflect health system performance regarding resource availability, infrastructure and access to healthcare [34,35]. Health interventions, including safe drinking water, improved child nutrition and breastfeeding and immunisation, have made progress in reducing under-5 mortality rates worldwide over the years, especially in African countries, where the highest mortality rates and lowest ECDI scores are reported [36].

Child survival, therefore, depends highly on environmental and population health outcomes. For instance, the LMICs with faster and prolonged improvements in basic public health and nutrition interventions have experienced a lowering of maternal and under-5 mortality rates over the years [36]. These interventions may enhance breastfeeding, safe drinking water, sanitation and hygiene, vitamin supplementation and immunisation coverage. Consequently, children may thrive and develop to their fullest potential under healthy conditions, which explains the linkage between ECD and the unfavourable health conditions related to mortality rates [36,37]. We ran an exploratory analysis to investigate the correlation between country-level mortality (maternal and under-5) rates and access to basic sanitation and drinking water. We found that countries with the highest mortality rates were more exposed to inadequate access to basic drinking water and sanitation (Pearson’s coefficient varying between −0.71 and −0.75) while having the highest poverty rates among children (60% of the countries within the highest 10% in terms of under-5 mortality had higher rates of extreme poverty) [38,39]. These factors pose a threat to proper development in early childhood [14,16,18,40], which is exacerbated in countries where high maternal illiteracy and poverty rates are also observed [31].

This is also in line with the results found for country income classification. Higher family income and wealth promote an improved home environment, favouring early stimulation aside from enhancements in children’s cognitive, social–behavioural and health status and parental commitment and wellbeing [19,20,29,32,41,42,43,44]. Income influences many different aspects linked to proper development during early childhood, including nurturing care. Early stimulation at the family home represents a supportive environment which improves child development outcomes in any of the following domains: sensory–motor, cognitive–language and social–emotional. As our results showed, more significant investments in the early years in books and ECE programs promote children’s skills. Previous studies have found that stimulating children’s brains early on through books and ECE programs yields much larger returns in the long term [45,46]. However, the proportion of children with developmental delay remains high. This is markedly unequal across most impoverished countries, especially in Sub-Saharan Africa, such as Chad, which has a prevalence of suspected delay of 67% [28,30]. Sociodemographic and economic characteristics at the regional and country level (Table S4) may also explain these results because of poorer access to clean water and sanitation and extreme poverty rates within countries in North and Sub-Saharan Africa and South Asia.

Furthermore, many studies [3,20,27] have demonstrated that stunting is one of the critical factors affecting ECD. However, we did not find any statistically significant association between stunting prevalence and ECD. This finding may be attributed to the nature of the data that are ecological along with the inclusion of other health-related variables, such as maternal and under-5 mortality, which have a strong correlation with stunting prevalence (Pearson’s coefficient = 0.62, and 0.63, respectively).

Surprisingly, a higher percentage of pregnant women receiving HIV treatment was negatively associated with ECD measures. This is likely because high HIV prevalence was almost perfectly correlated to the percentage of pregnant women receiving HIV treatment in our sampled countries. For instance, countries in the Sub-Saharan African region, such as Malawi, Uganda and Zimbabwe, reported the highest values regarding the level of access to HIV treatment for pregnant women, while they are also among the countries with the highest HIV prevalence in the world [47]. The highest HIV rates were observed in countries with the highest stunting prevalence and the lowest family income and ECD outcomes, which are likely reflected in the association found between the percentage of pregnant women receiving HIV treatment and ECDI. In addition, HIV-positive children, and children with HIV-positive parents, may experience disruption to their education and adequate development in the early years due to children’s absenteeism from educational activities caused by physical illnesses, medical appointments and financial losses incurred, among other factors [48,49].

However, our analysis has some limitations. It excludes cultural features and international conventions or national policies at the country level, including maternity and paternity leave, child protection and the conventions of breastmilk substitutes and child rights, due to a lack of data or null variability observed. Second, our study is a cross-sectional analysis and examines associations that indicate significant relationships but should not be interpreted as cause–effect. Third, the analysis includes 68 LMICs and may not necessarily represent all LMICs or specific regions due to limited data availability.

Future research should include the design of new potential interventions to model their effects at scale to enhance ECD outcomes in the most resource-constrained countries. Examples of evidence-driven strategies that can be implemented include improved access to clean water and sanitation in poor areas, along with nurturing care through multisectoral support of “tuition-free” pre-primary education programs [45]. From a government perspective, redistributive policies aimed at redressing social inequalities while investing in child health may influence ECD’s social and economic determinants in LMICs, as previous research has highlighted [22]. Moreover, longitudinal studies should be carried out to understand the causal effects of poor ECD determinants on children’s development over time, which remain unclear.

The design of new early childhood policies and implementing evidence-based practices are essential to construct and promote equality, healthy living and human development while fostering high economic returns in the longer term. Early years interventions may help reduce long-term social inequalities, resulting in government savings over the same timeframe. A continued increase in educational attainment throughout the life-course in some of the poorest countries is crucial to progress towards SDG 4, and other parallel targets aiming to reduce mortality and poverty and improve health and nutrition and gender equality [50,51]. Interventions should therefore aim to support family environments and caregivers by providing developmentally appropriate learning tools for young children from the most unequal and disadvantaged countries. Our results support the wider literature advocating for multisectoral action and collaboration (e.g., health, education, social development) to accelerate progress towards achieving the SDGs.

## 5. Conclusions

Our study’s findings show that maternal and child mortality, attendance at early childhood education programs, having books at home and income distribution within countries are associated with early child development outcomes among children aged 3–5 years. Greater maternal and under-5 mortality rates were negatively related to early childhood development. In contrast, higher country income and nurturing care indicators such as early childhood education attendance and children having books at home are positively associated with early child development outcomes. This study suggests that children from countries with high levels of maternal and under-5 mortality rates, high poverty rates derived from country income classification and inadequate early education and nurturing care levels are at higher risk of insufficient development during their early years. Nevertheless, other complex pathways should be accounted for to understand this difficult relationship. In the early years, investment to improve nurturing care and early childhood development outcomes are essential in achieving SDG targets.

## Figures and Tables

**Table 1:** Dependent and independent variables.

Variables	Description	Justification (Sign of the Expected Impact on Early Childhood Development; ECD)	Sources
**Dependent variables**			
Early Childhood Development Index (ECDI)	Index ranging from 0 to 100 that includes 10 items (set of questionnaires) in 4 early developmental domains: physical, language/cognition, approaches to learning and social-emotional skills. The higher the index, the more developed the children are in these domains.	Population-based measures indicating childhood development in children aged 3–5 years. These measures are essential to monitor and analyse the impact of interventions, population and ecological characteristics on early childhood development for decision- and policymaking purposes. [+]	[24,25] (see Supplementary Materials, Table S2).
ECDI 3 to 4 (%)	Percentage of children aged 36–59 months who were developmentally on track in three of the four domains of ECDI: literacy–numeracy, physical, social–emotional and learning.		[24,25] (see Supplementary Materials, Table S2).
**Independent variables**			
I. Nurturing care and early learning			
Attendance at Early Childhood Education (ECE) program	Percentage of children who attended an early childhood education program (pre-school).	Early childhood education programs aim to enhance and develop children’s critical skills (cognitive, socioemotional and motor). They have a protective impact against the future onset of disabilities and diseases and permit children to succeed in school. [+]	DHS 2012–2018, MICS 2010–2018, ICHS 2017 and Welfare Monitoring Survey 2015.
Early stimulation at home	Percentage of children with whom an adult has engaged in four or more activities related to reading books, telling stories, singing songs, going for a walk, playing games and spending time drawing or counting or naming with the child.	Children tend to learn more when they are interacting with others, especially with their parents and adults who teach them use different materials (and objects); therefore, children learn words, colors, shapes, numbers and general ideas. [+]	DHS 2012–2018, MICS 2010–2018, ICHS 2017 and Welfare Monitoring Survey 2015.
Children’s books at home (%)	Percentage of children who had three or more books at home.	Higher exposure to books and other written materials or narratives is essential to promote literacy and early language development in children. [+]	DHS 2012–2018, MICS 2010–2018, ICHS 2017 and Welfare Monitoring Survey 2015.
Children play games at home	Percentage of children aged 0–59 months who play with two or more of the following games at home: household objects or objects found outside (sticks, rocks, animals, shells, leaves, etc.), homemade toys or toys that came from a store.	Playing games is important for children’s cognitive expansion and exploration. When they play at home with their parents, they learn through the experience with those who orientate and teach them. [+]	DHS 2012–2018, MICS 2010–2018, ICHS 2017 and Welfare Monitoring Survey 2015.
II. Socioeconomic variables			
Gender inequality	Percentage ranging from 0 to 1. The higher the value, the greater the inequality.	Gender discrimination and inequality cause people to have limited autonomy in their rights and integrity, and to suffer the most from mental and physical health illnesses. As a consequence, there is a limited capability of decision making which impacts negatively on children’s growth, overall health status and proper brain development. [−]	United Nations Human Development Reports 2019 (available at http://hdr.undp.org/en/content/table-5-gender-inequality-index-gii, accessed on the 22nd of March 2021).
Income group	Three-level variable divided into low-income, low middle-income and upper middle-income countries. Higher values indicate upper income groups.	Children living in families from low-income backgrounds are more disadvantaged in terms of development and well-being because of living in poorer environments and conditions (including lack of sanitation and water). This affects children’s cognitive and other essential skills, and it negatively alters their behaviour. [+]	World Bank Income Classification (available online at https://data.worldbank.org/country/XO, accessed on the 22nd of March 2021).
Political stability	Index varying between −3 and 1.45. Higher values indicate the more stable countries are.	Politically stable countries provide more secure and stable environments where children could experience appropriate growth and stimulation. This may also include early childhood policies and programs oriented to nurturing, protection and easily accessible and affordable childcare, as well as consistent follow-up throughout children’s early years which aims to control their health status. [+]	World Bank worldwide governance indicators 2018 (available online at https://databank.worldbank.org/source/worldwide-governance-indicators, accessed on the 22nd of March 2021).
Net migration rate per 1000 population	Continuous variable ranging between -24 and 22. Higher values indicate greater migration.	Even though migration may allow children to have opportunities and access to education and health services, it can pose challenges including discrimination, marginalization and barriers to accessing basic services. Moreover, high immigration enforcement levels may negatively impact children’s development in the short and long run, affecting their mental health and equitable development. [+ or −]	World Population Prospects: The 2019 Revision (available online at https://data.un.org/Data.aspx?d=PopDiv&f=variableID%3A85, accessed on the 22nd of March 2021).
III. Health and mortality-related variables			
Under-5 stunting	Prevalence (%) of stunting in children under 5 years of age.	Stunting in children is linked to low nutrition, high probability of suffering from health illnesses, poor educational performance and cognition and several other effects appearing in late life, such as low adult earnings, excessive weight gain and high risks of having chronic diseases, among others. [−]	DHS 2012–2018, MICS 2010–2018, ICHS 2017 and Welfare Monitoring Survey 2015.
Low birthweight	Percentage of children with low birthweight.	Low birth at weight is a consequence of poor antenatal care which causes poor childhood development in the first years, including disabilities and developmental delays. [−]	UNICEF/WHO Low birthweight (LBW) estimates, 2019 Edition.
Pregnant woman receiving HIV treatment	Percentage of pregnant women receiving HIV treatment (%).	Pregnant women living with HIV who receive treatment are less likely to transmit the disease to their babies, safeguarding their health status. [−]	Global AIDS Monitoring and UNAIDS 2019 estimates.
Maternal mortality	Continuous variable measured as a rate per 100,000 live births.	Greater maternal mortality, especially when children are younger, increases the risk of suffering from a deplorable health status (including morbidities), and poor early childhood development. [−]	WHO, UNICEF, UNFPA, World Bank Group and UNPD (MMEIG)—September 2019.
Under-5 mortality	Continuous variable measured as a rate per 1000 live births.	Higher values of under-5 mortality indicate the country has made less investments in adequate nutrition and food quality, immunisation and safe water and sanitation which lead to preterm birth, or simply birth complications including asphyxia, trauma, diarrhoea and pneumonia, among other diseases. [−]	UN Inter-agency Group for Child Mortality Estimation (UN IGME) in 2019.

Notes: Specific information on the year of the sources is available upon request. Most information can be found online at https://nurturing-care.org/resources/country-profiles/ (accessed on the 22nd of March 2021). DHS stands for Demographic and Health Survey, whereas MICS stands for Multiple Indicator Cluster Survey. We carried out a cross-check analysis between both surveys (MICS and DHS), using estimates of stunting prevalence as an example for Zimbabwe and the Dominican Republic. We used MICS 2014 and DHS 2015 for the former and MICS 2014 and DHS 2013 for the latter. No significant difference was found between MICS and DHS estimates for stunting prevalence in children aged 3–5 years (MICS = 27.03%, DHS = 26.8%; MICS = 7.12%, DHS = 6.9%, respectively).

**Table 2:** Number of countries included by the World Health Organization (WHO) region and World Bank income group.

Region	Number of Countries
East Asia and Pacific	9
Europe and Central Asia	11
Latin America and Caribbean	15
Middle East and North Africa	5
South Asia	4
Sub-Saharan Africa	24
**World Bank income group**	**Number of countries**
Low income	17
Lower middle income	25
Upper middle income	26

**Table 3:** Descriptive statistics (*N* = 68 low-and-middle income countries (LMICs)).

Description	Mean	Median	SD	IQR
**Dependent variables**				
Early Childhood Development Index (ECDI)	73.48	73.40	14.80	24.35
% of children developmentally on track	72.58	74.10	14.50	22.03
**Health related data**				
Under-5 mortality rate per 1000 live births	38.53	28.50	31.84	37.50
Maternal mortality rate per 100,000 live births	248.31	129.50	277.65	355.50
% of children low birthweight	11.33	11.09	4.73	6.89
% of children under-five stunting	21.46	20.14	13.20	23.17
Treatment for HIV+ pregnant women (%)	71.27	79.05	22.17	31.25
**Nurturing care and early education**				
Early stimulation at home (%)	65.13	67.35	22.38	41.70
Children’s books in the home (%)	23.49	10.90	25.51	43.05
Children play games at home (%)	54.30	55.90	13.94	18.40
Attendance at early childhood education program (%)	35.84	30.49	24.76	40.75
**Socioeconomic variables**				
Political Stability Index 2018	−0.40	−0.33	0.81	0.78
Gender inequality	44.96	46.14	14.66	21.38
Income category	1.13	1.00	0.79	1.50
Net migration rate (per 1000 population)	−0.90	−0.52	3.93	2.27
**Auxiliary variables**				
GDP pp 2018 in USD	3962.75	3147.02	3447.41	4838.55
Human Development Index 2018	64.03	66.85	12.24	21.10
Population 2019 in millions	2.5e^7^	9.6e^6^	4.8e^7^	1.9e^7^

Notes: SD: standard deviation. IQR: interquartile range (75th percentile–25th percentile). e stands for the exponential function.

**Table 4:** Linear regression results (*N* = 68 low- and middle-income countries (LMICs)).

Independent Variables	Model 1			Model 2		
	ECDI Index			ECDI 3 out of 4 Domains		
	*β*	95% CI	*p*-Value	*β*	95% CI	*p*-Value
Under-5 stunting	0.01	−0.23, 0.25	0.91	−0.01	−0.26, 0.23	0.93
Low birthweight	−0.43 *	−0.93, 0.06	0.08	−0.19	−0.67, 0.29	0.42
Pregnant woman receiving HIV treatment	−0.16 ***	−0.25, −0.07	<0.001	−0.14 ***	−0.24, −0.05	<0.001
Gender inequality	0.06	−0.22, 0.33	0.69	0.05	−0.24, 0.34	0.73
Income group (REF: low-income country)						
Lower middle-income country	0.89	−4.29, 6.07	0.73	2.61	−3.13, 8.34	0.37
Upper middle-income country	7.17 **	0.35, 13.99	0.04	8.33 **	1.45, 15.21	0.02
Political stability	1.29	−1.02, 3.61	0.27	0.54	−1.76, 2.84	0.64
Migration rate	−0.05	−0.39, 0.30	0.79	−0.08	−0.46, 0.31	0.69
Maternal mortality	−0.02 ***	−0.03, −0.01	0.01	−0.02 ***	−0.03,−0.00	0.01
Under-5 mortality	−1.42 ***	−2.25, −0.59	<0.001	−1.60 ***	−2.47, −0.74	<0.001
Nurturing care and early learning						
Attendance at ECE program	0.12 **	0.03, 0.22	0.01	0.11 **	0.03, 0.20	0.01
Early stimulation at home	−0.13	−0.30, 0.03	0.11	−0.11	−0.28, 0.06	0.19
Children’s books at home	0.14 **	0.00, 0.27	0.04	0.09	−0.06, 0.24	0.23
Children play games at home	−0.10	−0.25, 0.05	0.18	−0.11	−0.27, 0.06	0.20
Constant	112.43 ***	91.14, 133.71	<0.001	109.55 ***	86.58, 132.53	<0.001
R^2^	86%			85%		
F-test (*p*-value)	29.15 (*p* < 0.001)				22.13 (*p* < 0.001)	
AIC	456.02			458.66		
VIF	3.50				3.58	
Breusch–Pagan before robust SE	6.00 (*p* = 0.0143)			8.19 (*p* = 0.0042)		
Heteroscedasticity	Chi^2^ = 68, *p*-value = 0.44			Chi^2^ = 68, *p*-value = 0.44		
Skewness	Chi^2^ = 10, *p*-value = 0.59			Chi^2^ = 11, *p*-value = 0.54		
Kurtosis	Chi^2^ = 0.9, *p*-value = 0.64			Chi^2^ = 0.29, *p*-value = 0.59		

Notes: * *p* < 0.1, ** *p* < 0.05, *** *p* < 0.01, robust standard errors were used as suggested by Breusch–Pagan test. Akaike Information Criterion: AIC. Variance Inflation Factor: VIF. Early Childhood Development Index (ECDI) was normally distributed (Supplementary Materials, Figure S1).

## Data Availability

Data are shared publicly at the WHO, the WB, the DHS or the UN websites/repositories.

## Supplementary Materials

The following are available online at https://www.mdpi.com/1660-4601/18/7/3340/s1, Figure S1: Kernel density estimate for the ECDI index and percentage of children developmentally on track, Figure S2: Bivariate correlation table, Figure S3: Residual’s normality plot, Table S1: List of countries (N = 68 LMICs), Table S2: ECDI specific data sources, Table S3: Missing data, Table S4: Main sociodemographic and economic indicators by World Bank Region.
