# Supplementary material for: Exploring the Associations between Early Childhood Development Outcomes and Ecological Country-Level Factors across Low- and Middle-Income Countries
Source: Int J Environ Res Public Health. 2021 Mar 24;18(7):3340. doi: 10.3390/ijerph18073340 (PMC8037361; doi:10.3390/ijerph18073340)
Supplement: Supplementary file 1 [file ijerph-18-03340-s001.pdf]

Exploring the associations between early childhood development outcomes and ecological country-level factors in low-and-middle income countries

Supplementary material

Table A1. List of countries (N=68 LMICs)

Country name	Country abbreviation	Country name	Country abbreviation
Algeria	DZA	Kazakhstan	KAZ
Argentina	ARG	Kyrgyzstan	KGZ
Bangladesh	BGD	Lao People's Democratic Republic	LAO
Belarus	BLR	Malawi	MWI
Belize	BLZ	Maldives	MDV
Benin	BEN	Mali	MLI
Bhutan	BTN	Marshall Islands	MHL
Bosnia and Herzegovina	BIH	Mauritania	MRT
Burundi	BDI	Mexico	MEX
Cambodia	KHM	Mongolia	MNG
Cameroon	CMR	Montenegro	MNE
Central African Republic	CAF	Nepal	NPL
Chad	TCD	Nigeria	NGA
Congo	COG	North Macedonia	MKD
Costa Rica	CRI	Panama	PAN
Cote D'Ivoire	CIV	Paraguay	PRY
Cuba	CUB	Republic of Moldova	MDA
Democratic People's Republic of Korea	PRK	Rwanda	RWA
Democratic Republic of the Congo	COD	Saint Lucia	LCA
Dominican Republic	DOM	Sao Tome and Principe	STP
El Salvador	SLV	Senegal	SEN
Eswatini	SWZ	Serbia	SRB
Gambia	GMB	Sierra Leone	SLE
Georgia	GEO	State of Palestine	PSE
Ghana	GHA	Suriname	SUR
Guinea	GIN	Thailand	THA
Guinea-Bissau	GNB	Timor-Leste	TLS
Guyana	GUY	Togo	TGO
Haiti	HTI	Tunisia	TUN
Honduras	HND	Turkmenistan	TKM
Indonesia	IDN	Uganda	UGA
Iraq	IRQ	Ukraine	UKR
Jamaica	JAM	Vietnam	VNM
Jordan	JOR	Zimbabwe	ZWE

Notes: Data available at https://www.unicef.org/earlychildhood/index_69536.html.

Table A2. ECDI specific data sources (N=68 LMICs)

CC	Data source	CC	Data source
DZA	MICS 2012-13	KAZ	MICS 2015
ARG	MICS 2011-12	KGZ	MICS 2014
BGD	MICS 2012-13	LAO	MICS 2017
BLR	MICS 2012	MWI	MICS 2013-14
BLZ	MICS 2015	MDV	DHS 2016-17
BEN	DHS 2017-18	MLI	MICS 2015
BTN	MICS 2010	MHL	ICHNS 2017
BIH	MICS 2011-12	MRT	MICS 2015
BDI	DHS 2016-17	MEX	MICS 2015
KHM	DHS 2014	MNG	MICS 2013
CMR	MICS 2014	MNE	MICS 2013
CAF	MICS 2010	NPL	MICS 2014
TCD	DHS 2014-15	NGA	MICS 2016
COG	MICS 2014-15	MKD	MICS 2011
CRI	MICS 2011	PAN	MICS 2013
CIV	MICS 2016	PRY	MICS 2016
CUB	MICS 2014	MDA	MICS 2012
COD	DHS 2013-14	RWA	DHS 2014-15
PRK	MICS 2017	LCA	MICS 2012
DOM	MICS 2014	STP	MICS 2014
SLV	MICS 2014	SEN	DHS 2017
SWZ	MICS 2014	SRB	MICS 2014
GMB	MICS 2010	SLE	MICS 2017
GEO	WMS 2015	PSE	MICS 2014
GHA	MICS 2011	SUR	MICS 2010
GIN	MICS 2016	THA	MICS 2015-16
GNB	MICS 2014	TLS	DHS 2016
GUY	MICS 2014	TGO	DHS 2013-14
HTI	DHS 2016-17	TUN	MICS 2011-12
HND	DHS 2011-12	TKM	MICS 2015-16
IDN	RISKESDAS 2018	UGA	DHS 2016
IRQ	MICS 2018	UKR	MICS 2012
JAM	MICS 2011	VNM	MICS 2014
JOR	DHS 2017-18	ZWE	MICS 2014

Notes: CC stands for Country code. DHS is for Demographic and Health Survey, whereas MICS is for Multiple Indicator Cluster Survey. More specific information can be found elsewhere (https://www.unicef.org/earlychildhood/index_69536.html). Information on the % of children developmentally on track can be found at https://data.unicef.org/resources/data_explorer/unicef_f/?ag=UNICEF&df=GLOBAL_DATAFLOW&ver=1.0&dq=.ECD_CHLD_36-59M_LMPSL..&startPeriod=2016&endPeriod=2020. Data collection was similar across every country. It consisted of a random sample accounting for urban and rural areas for each region within countries. The only country where only urban populations were included (municipalities with a population of more than 5,000) was Argentina as the country's rural population is scattered and accounts for less than 10 per cent of the total population. However, sampling weights were used to make the estimates consistent with the real population to report national level data. ECDI measures were consistent over time even though we extracted ECDI values from different MICS surveys (MICS4, MICS5 and MICS6). We also restricted the sample to the youngest children aged 36-59 months as this information was available for all countries.

Table A3. Missing data (N=68 LMICs)

**Description**	**Missing data (N)**	**Missing data (%)**	**Used as control** (T-test, p-value) [A]
Population in 2019 in millions		0.00%	
Number of annual births (in millions) **	1	1.47%	
Number of children under the age of 5 in 2019 (in millions) **	1	1.47%	
% of children under the age of 5 in 2019 (in millions) **	1	1.47%	
Under-5 mortality rate per 1,000 live births		0.00%	
Maternal mortality rate per 100,000 live births	1	1.47%	(0.01, 0.98)
% of Preterm births **	1	1.47%	
% of children with low birthweight	12	17.65%	(-0.48, 0.63)
% of children under-five stunting		0.00%	
% of Children living in poverty **	30	44.12%	
% of children in violent discipline **	10	14.71%	
% of children under inadequate supervision **	1	1.47%	
% of young mothers (births by 18 years of age) **	6	8.82%	
(%) Composite burden 2005 **	23	33.82%	
(%) Composite burden 2010 **	23	33.82%	
(%) Composite burden 2015 **	23	33.82%	
Risks by gender girls (%) **	32	47.06%	
Risks by gender boys (%) **	32	47.06%	
Risks by residence rural (%) **	32	47.06%	
Risks by residence urban (%) **	32	47.06%	
Lifetime cost of growth deficit in early childhood **	7	10.29%	
**Early Childhood Development Index (ECDI)**		**0.00%**	
**% of children developmentally on track**		**0.00%**	
Antenatal care (%) **		0.00%	
Pregnant women receiving treatment for HIV (%)	19	27.94%	(-0.11, 0.91)
Postnatal visits (%) **	14	20.59%	
Care-seeking for child pneumonia (%) **	2	2.94%	
Early initiation of breastfeeding (%) **		0.00%	
Exclusive breastfeeding (%) **		0.00%	
Minimum acceptable diet (%) **	18	26.47%	
Early stimulation at home (%)	1	1.47%	(-0.03, 0.97)
Children's books in the home (%)	1	1.47%	(0.07, 0.94)
Playthings at home (%)	1	1.47%	(-0.02, 0.98)
Attendance in early childhood education (%)		0.00%	
Birth registration (%) **		0.00%	
Positive discipline (%) **	11	16.18%	
Basic drinking water (%) **		0.00%	
Basic Sanitation (%) **		0.00%	
GDP pp 2018 in USD		0.00%	
Political stability index 2018		0.00%	
Human Development Index 2018	1	1.47%	(0.07, 0.94)
Control of Corruption Index 2018 **	1	1.47%	
Gender Inequality	8	11.76%	(-0.17, 0.86)
Child and family social protection **	1	1.47%	
National minimum wage **	11	16.18%	
Paid maternity leave **	4	5.88%	
Paid paternity leave **	2	2.94%	
International Code of Marketing of Breastmilk Substitutes **	1	1.47%	
Migration rate	2	2.94	(-0.11, 0.91)

Notes: Variables highlighted in green in the fourth column were used as controls for the imputation process. ** Variables were tested but not included in the final model. [A] Two-sample t-test were calculated between imputed and original values.

Table A4. Main sociodemographic and economic indicators by World Bank Region

INDICATOR/WB Region	AFRICA	EAST ASIA AND PACIFIC	EUROPE AND CENTRAL ASIA	LATIN AMERICA AND THE CARIBBEAN	MIDDLE EAST AND NORTH AFRICA	SOUTH ASIA
Total population (millions)	1,106	2,094	399	614	389	1,836
Population growth (annual %)	2.7	0.6	0.5	0.9	1.7	1.2
GNI per capita (Atlas method, current US$)	1,536	8,299	8,014	8,355	3,861	2,019
GDP per capita growth (annual %)	(0.4)	5.2	1.2	(0.1)	1.2	3.6
Population living below $1.90 a day (millions)	420	28	6	28	28	275
Life expectancy at birth, females (years)	63	78	78	78	76	71
Life expectancy at birth, males (years)	60	73	70	72	72	68
Carbon dioxide emissions (megatons)	848	11,421	2,979	1,658	1,475	2,737
Extremepoverty(% population below $1.90 a day, 2011 PPP)	42.3	1.3	1.2	4.4	7.2	16.1
Vulnerable employment, total (% of total employment) (modelled ILO estimate)	74	47	18	34	31	70
Under-5 mortality rate per 1,000 live births	78	16	13	17	24	42
Primary completion rate (% relevant age group)	69	98	97	99	90	92
Individuals using the Internet (% of population)	25	51	73	66	60	30
Access to electricity (% population)	48	98	100	98	96	92
People using at least basic sanitation (%)	31	82	94	86	89	59
People using at least basic drinking water services (%)	61	92	96	96	93	92

Notes: ILO = International Labor Organization; PPP = purchasing power parity. https://www.worldbank.org/en/about/annual-report/region-perspectives. GNI stands for Gross National Income.

a. The most current data available between 2013 and 2019; visit http://data.worldbank.org for data updates.

b. 2002 data. For poverty estimates, see the regional groups on http://iresearch.worldbank.org/PovcalNet/data.aspx.

Figure A1. Kernel density estimate for the ECDI index and percentage of children developmentally on track (N=68 LMICs)

a)

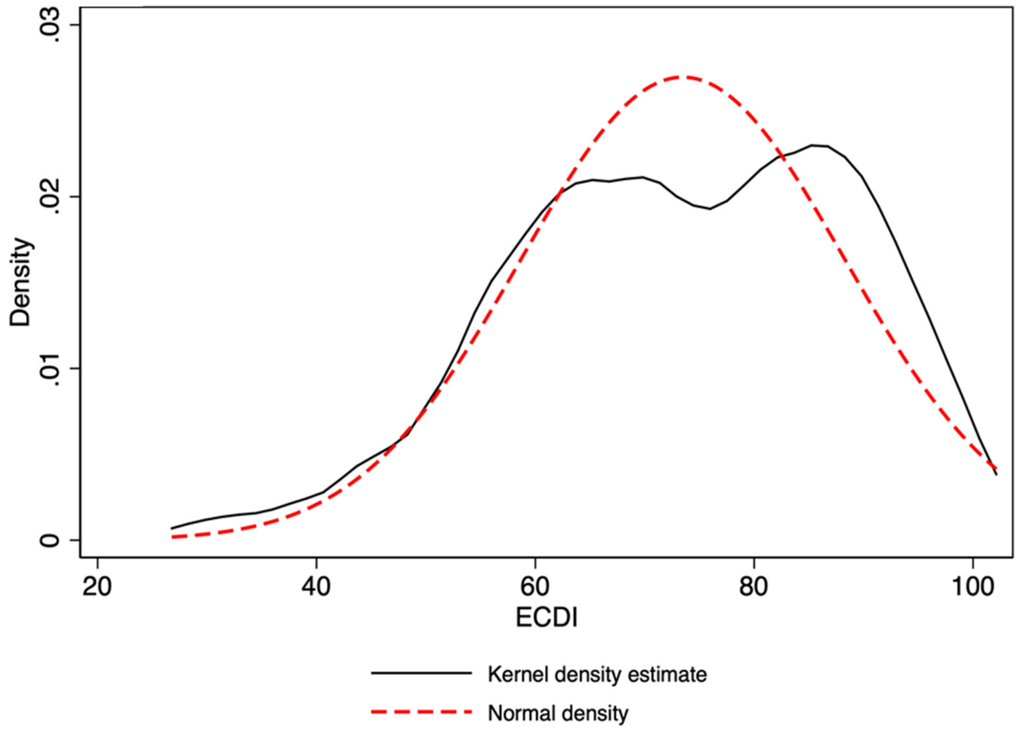

b)

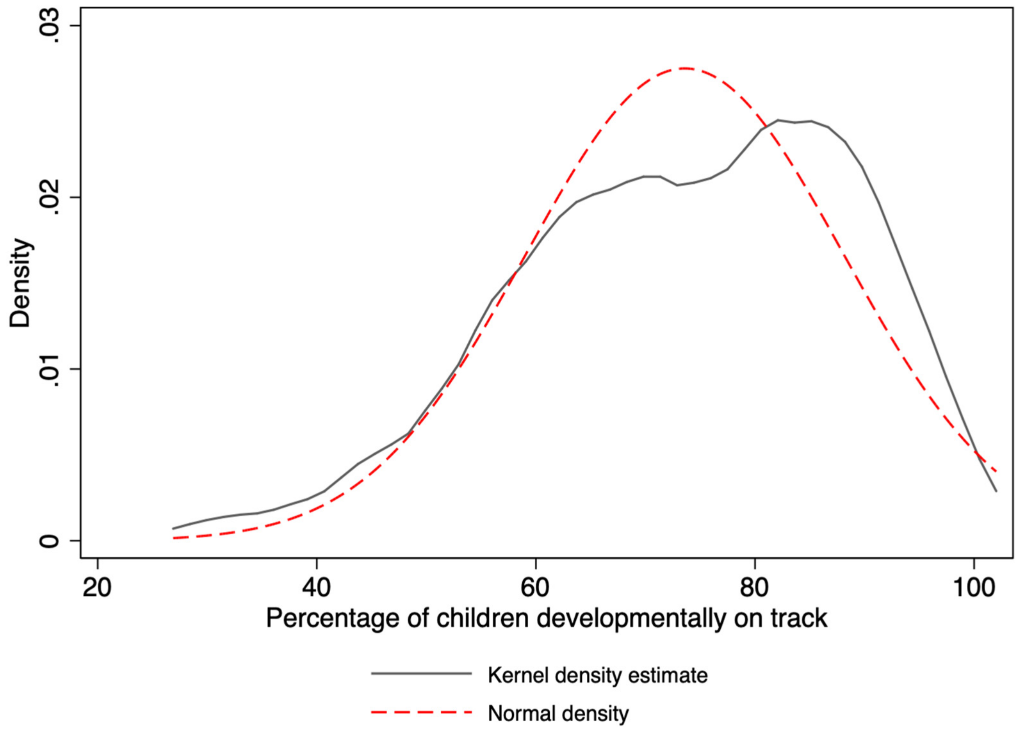

Figure A2. Bivariate correlation table (N=68 LMICs)

ECDI	1.000	0.986	-0.678	0.605	-0.794	0.671	-0.835	-0.778	-0.008	0.372	0.184	0.783	0.722	0.511	-0.474
ECD (3 of 4 categories %)	0.986	1.000	-0.670	0.587	-0.775	0.669	-0.844	-0.781	-0.020	0.355	0.162	0.758	0.731	0.517	-0.421
Under 5 Stunting %	-0.678	-0.670	1.000	-0.559	0.621	-0.537	0.628	0.634	-0.204	-0.316	-0.050	-0.666	-0.699	-0.533	0.491
Attendance to ECE %	0.605	0.587	-0.559	1.000	-0.493	0.527	-0.589	-0.474	0.342	0.355	0.250	0.683	0.390	0.408	-0.268
Gender Inequality Index	-0.794	-0.775	0.621	-0.493	1.000	-0.727	0.825	0.788	-0.105	-0.362	-0.132	-0.825	-0.657	-0.520	0.623
Early Stimulation at Home %	0.671	0.669	-0.537	0.527	-0.727	1.000	-0.742	-0.635	-0.033	0.142	0.149	0.807	0.627	0.532	-0.458
Children Under 5 mortality	-0.835	-0.844	0.628	-0.589	0.825	-0.742	1.000	0.750	-0.106	-0.361	-0.128	-0.812	-0.675	-0.610	0.375
Maternal mortality	-0.778	-0.781	0.634	-0.474	0.788	-0.635	0.750	1.000	-0.119	-0.418	-0.101	-0.614	-0.663	-0.547	0.412
Treatment HIV for pregnant Women	-0.008	-0.020	-0.204	0.342	-0.105	-0.033	-0.106	-0.119	1.000	0.330	-0.007	0.132	0.067	0.069	-0.152
Political Stability	0.372	0.355	-0.316	0.355	-0.362	0.142	-0.361	-0.418	0.330	1.000	0.052	0.218	0.318	0.252	-0.087
Migration	0.184	0.162	-0.050	0.250	-0.132	0.149	-0.128	-0.101	-0.007	0.052	1.000	0.213	0.099	-0.091	-0.048
Children have books at home	0.783	0.758	-0.666	0.683	-0.825	0.807	-0.812	-0.614	0.132	0.218	0.213	1.000	0.624	0.513	-0.548
Income group	0.722	0.731	-0.699	0.390	-0.657	0.627	-0.675	-0.663	0.067	0.318	0.099	0.624	1.000	0.553	-0.468
Children playing things at home	0.511	0.517	-0.533	0.408	-0.520	0.532	-0.610	-0.547	0.069	0.252	-0.091	0.513	0.553	1.000	-0.306
Low birthweight %	-0.474	-0.421	0.491	-0.268	0.623	-0.458	0.375	0.412	-0.152	-0.087	-0.048	-0.548	-0.468	-0.306	1.000

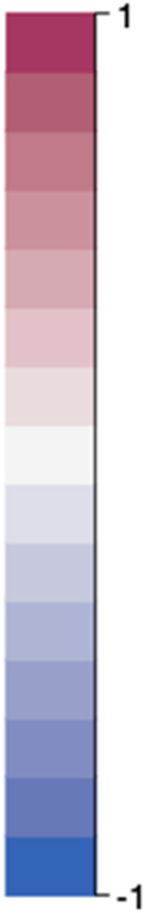

Figure A3. Residuals normality plot (N=68 LMICs)

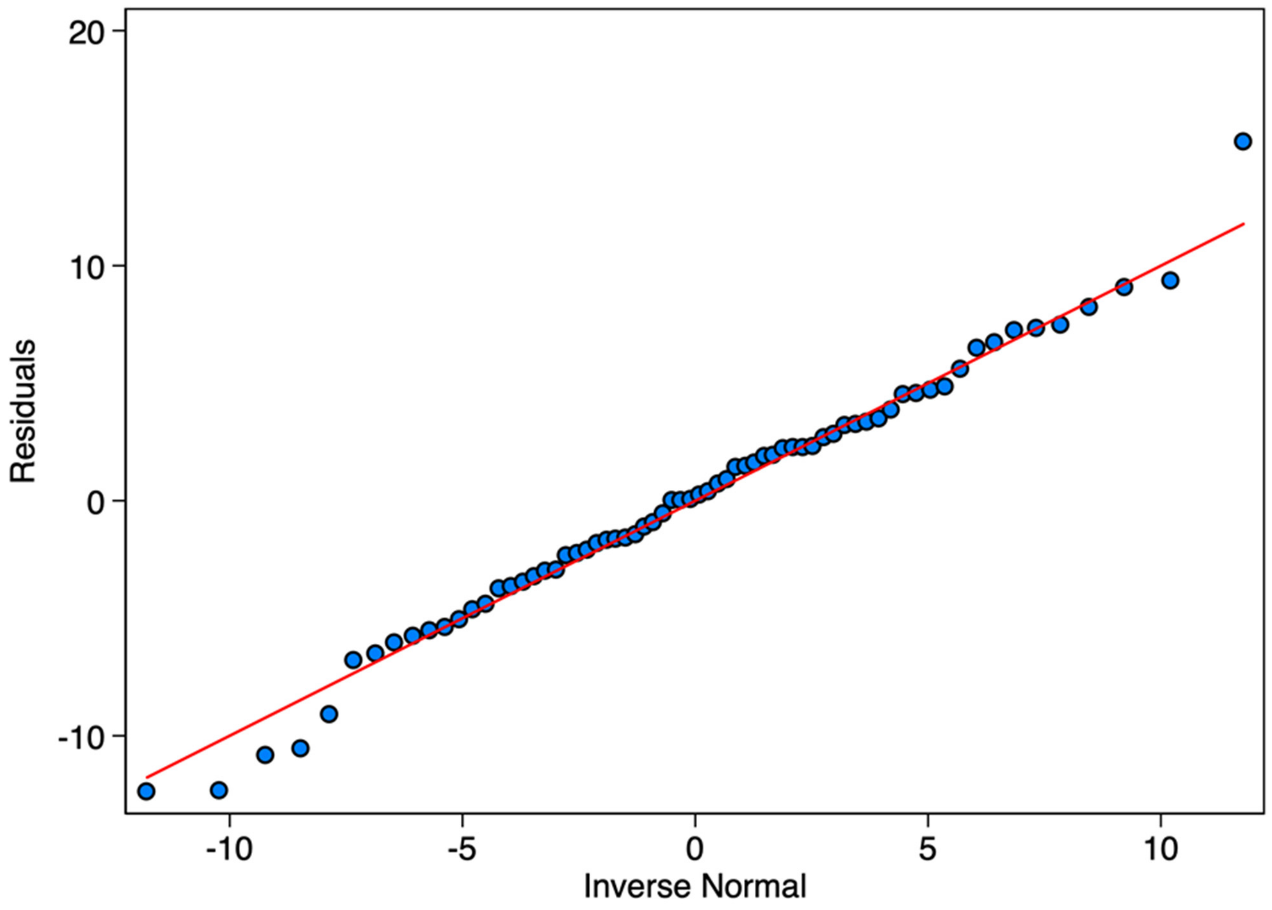

Notes: Blue circles stand for the residuals of Model 1, Table 4.
